# Acute lung injury by gastric fluid instillation: activation of myofibroblast apoptosis during injury resolution

**DOI:** 10.1186/s12931-018-0763-6

**Published:** 2018-04-10

**Authors:** Pedro Ayala, Jorge Torres, Raúl Vivar, Manuel Meneses, Pablo Olmos, Tamara San Martin, Gisella R. Borzone

**Affiliations:** 1 Department of Respiratory Diseases and Medical Research Center, Pontificia Universidad Católica de Chile, Marcoleta 350, piso 1, Santiago, Chile; 2 Department of Diabetes and Nutrition, Pontificia Universidad Católica de Chile, Santiago, Chile; 3 Pathology Unit, Instituto Nacional del Tórax, Santiago, Chile

**Keywords:** Apoptosis, Lung injury resolution, Aspiration, Myofibroblast, MMP-2/TIMP-2 balance

## Abstract

**Background:**

Gastric contents aspiration in humans has variable consequences depending on the volume of aspirate, ranging from subclinical pneumonitis to respiratory failure with up to 70% mortality. Several experimental approaches have been used to study this condition. In a model of single orotracheal instillation of gastric fluid we have shown that severe acute lung injury evolves from a pattern of diffuse alveolar damage to one of organizing pneumonia (OP), that later resolves leaving normal lung architecture. Little is known about mechanisms of injury resolution after a single aspiration that could be dysregulated with repetitive aspirations. We hypothesized that, in a similar way to cutaneous wound healing, apoptosis may participate in lung injury resolution by reducing the number of myofibroblasts and by affecting the balance between proteases and antiproteases. Our aim was to study activation of apoptosis as well as MMP-2/TIMP-2 balance in the sub-acute phase (4–14 days) of gastric fluid-induced lung injury.

**Methods:**

Anesthesized Sprague-Dawley rats received a single orotracheal instillation of gastric fluid and were euthanized 4, 7 and 14 days later (*n* = 6/group). In lung tissue we studied caspase-3 activation and its location by double immunofluorescence for cleaved caspase-3 or TUNEL and alpha-SMA. MMP-2/TIMP-2 balance was studied by zymography and Western blot. BALF levels of TGF-β_1_ were measured by ELISA.

**Results:**

An OP pattern with Masson bodies and granulomas was seen at days 4 and 7 that was no longer present at day 14. Cleaved caspase-3 increased at day 7 and was detected by immunofluorescence in Masson body-alpha-SMA-positive and –negative cells. TUNEL-positive cells at days 4 and 7 were located mainly in Masson bodies. Distribution of cleaved caspase-3 and TUNEL-positive cells at day 14 was similar to that in controls. At the peak of apoptosis (day 7), an imbalance between MMP-2 activity and TIMP-2 expression was produced by reduction in TIMP-2 expression.

**Conclusions:**

Apoptosis is activated in Masson body-alpha-SMA–positive and –negative cells during the sub-acute phase of gastric fluid-induced lung injury. This mechanism likely contributes to OP resolution, by reducing myofibroblast number and new collagen production. In addition, pre-formed collagen degradation is favored by an associated MMP-2/TIMP-2 imbalance.

## Background

Gastric contents aspiration is a high-risk condition for lung injury. Consequences are highly variable and range from subclinical pneumonitis to respiratory failure. Reported prevalence in at risk patients can range from 10 to 70%, whereas mortality has been reported to be as high as 70% depending on the volume of the aspirate [1].

Several approaches have been used in experimental animals to model this condition and have provided insight into the pathogenesis of aspiration-induced lung injury [2–6]. Our group has developed a rat model of single orotracheal instillation of whole gastric contents [7] to study the time course of morphological and biochemical changes during injury and resolution and, has found that acute lung injury (ALI) evolves from an initial pattern of diffuse alveolar damage with severe derangement of the alveolar-capillary barrier and an intense inflammatory and hemorrhagic infiltration, to one of organizing pneumonia (OP). As initial markers of ALI resolve, an organization process ensues, involving intraluminal plugs of myofibroblasts and collagen fibers affecting peribronchiolar alveolar spaces. This OP pattern arises as a way of organization of the early intense inflammatory exudate that characterizes aspiration-induced ALI in the rat, and shows complete resolution at day 60 after instillation [7]. Unlike what has been shown in a small proportion of patients with aspiration who develop progressive fibrosis, intra-alveolar fibrosis in our model is completely reversible.

Little is known about mechanisms involved in injury resolution after gastric contents aspiration or after other insults. Despite its important clinical role, the biological processes involved in the prompt and orderly elimination of the intra-alveolar fibrosis are unknown.

Given the fact that the continuum of alterations observed in aspiration-induced lung injury shares significant similarities with the phases described in normal wound healing [8], we speculate that mechanisms involved in injury resolution in cutaneous wound healing may, at least in part, be involved in resolution of lung injury in this model. In fact, in wound healing, once acute injury markers resolve, myofibroblasts are attracted into the wound and synthesize extracellular matrix (ECM) components that constitute the wound granulation tissue. These changes are followed by both: a) matrix metalloproteinase-dependent mechanisms contributing to the remodeling of the ECM [8, 9] and, b) a significant reduction in cellularity, due to the elimination of collagen-producing myofibroblasts, which is a prerequisite for sustained fibrosis resolution. In different fibrotic conditions myofibroblast removal has been shown to occur through several alternative cell fates such as apoptosis, senescence, and dedifferentiation [10].

Apoptosis has been shown to be the primary mechanism of myofibroblast elimination in granulation tissue during the resolution phase of normal wound repair [11]. This controlled process of cell death is characterized by both DNA fragmentation and nuclear shrinkage and exhibits none or minimal inflammatory response. It is triggered by specific stimuli and is carried out by intracellular pathways [12]. In ALI, apoptosis has been studied mainly in the initial phases of the process. Studies have focused on apoptosis of inflammatory [13] and alveolar epithelial cells [14–17] during the acute phase of increased permeability of the alveolar-capillary membrane. There is no information about the role of apoptosis of cells that are part of the phase of organization into the OP pattern (namely myofibroblasts) in our model. Since the reduction in myofibroblast number is not in itself sufficient to allow for remodeling of the existing excess collagen, apoptosis in wound healing has been shown to be associated with changes in the matrix metalloproteinases/tissue inhibitor of metalloproteinases (MMPs/TIMPs) balance toward an increased ECM degradation activity [8, 9].

We hypothesized that in the resolution of the organizing pneumonia induced by gastric contents aspiration, both apoptosis of myofibroblasts and upregulation of matrix degradation will be involved, the later related to a shift in the MMPs/TIMPs balance toward an increased degradation activity.

Our aim was to study activation of apoptosis and the MMPs/TIMPs balance during the subacute phase of lung injury induced by a single instillation of gastric fluid.

For this purpose, lung tissue of rats instilled orotracheally with a single dose of gastric fluid was studied at days 4, 7 and 14 for evidence of apoptosis by means of terminal deoxynucleotidyl transferase (TdT)-mediated deoxyuridine triphosphate nick-end labeling (TUNEL) as well as by caspase activation in α-SMA-positive cells present in intra-alveolar fibrosis. Zymography was used to study MMP-2 activity and TIMP-2 expression was studied by western blot analysis.

We found that activation of apoptosis during the repair phase of aspiration-induced ALI contributes to the disappearance of cells that are present in the fibroproliferative response. We also found that an imbalance between MMP-2 activity and TIMP-2 expression is also established by reduction in TIMP-2 expression, contributing to the removal of non-cellular components of the fibroproliferative response.

## Methods

The study was performed according to a protocol submitted to and approved by the Animal Research Ethics Committee of the Pontificia Universidad Católica de Chile in adult male Sprague-Dawley rats (270–300 g, 55–65 days old).

**Rat model of orotracheal instillation of gastric fluid:**

**Fig. 1:**
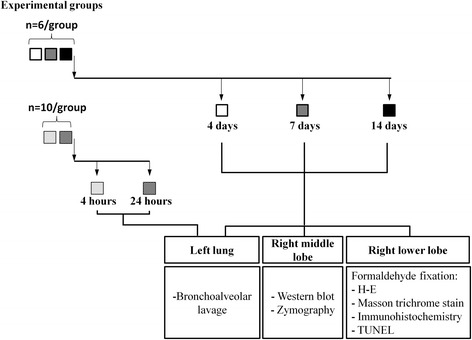
Experimental design. Diagram showing animal groups, instillation schedule and timing of animal sacrifice as well as sampling and analysis

- **Gastric contents pool:** Adult male Sprague-Dawley rats fasted overnight were anesthetized i.p. with xylazine-ketamine (5.1 and 55.1 mg/kg, respectively) to obtain gastric fluid through a gastrotomy. Gastric fluid samples were pooled, filtered through a 100 um mesh, and kept at −80 °C. Animals were euthanized thereafter by exsanguination under anesthesia.
- **Orotracheal instillation of gastric fluid:** Under the same anesthetic protocol, another set of animals was orotracheally intubated with a 22 gauge wire-fed catheter. Visualization of the glottis was achieved using a modified human otoscope (Welch Allyn, Skaneateles Falls, NY). A volume of gastric fluid previously determined by the authors (data not shown) to distribute evenly (1.5 mL/kg, pH 1.69) was instilled, and animals were allowed to recover spontaneously from anesthesia.
- **Study groups:** Fig. 1 illustrates the study protocol and tissue sampling diagram. Histological and biochemical studies were performed at days 4, 7 and 14 after instillation (*n* = 6 per group). Another set of animals was used to study levels of TGF-β1 in bronchoalveolar lavage (BAL) at earlier times: 4 h (*n* = 10) and 24 h (*n* = 10). Animals without intervention (*n* = 6) served as controls because only a negligible change in the proportion of alveolar cells was seen in saline-treated animals.

### Sample collection

Lungs were excised *en bloc*, and the left main bronchus was cannulated for BAL. For each animal, three aliquots of 0.15 M saline (1 mL each) were instilled, immediately aspirated, pooled, and stored at − 80 °C for cytokine levels determination. The right upper and middle lobes were excised and frozen for biochemical and molecular analysis. The right lower lobe was fixed at 20 cm H_2_O with 10% buffered formaldehyde solution and paraffin embedded for histological studies.

### Histologic evidence of tissue injury

For each animal, four right lower lobe longitudinal sections were embedded in paraffin, sectioned at 5 μm, and stained with hematoxylin-and-eosin and Masson’s trichrome for analysis by light microscopy.

### Immunohistochemistry for alpha-smooth muscle actin (α-SMA)

Abundance of myofibroblasts was evaluated using immunohistochemistry for α-SMA by the immunoperoxidase technique [18]. Endogenous peroxidase activity in lung sections was blocked with 3% H_2_0_2_ for 10 min. Nonspecific reactivity was blocked with horse serum for 10 min. Samples were then incubated with a mouse monoclonal anti-α-SMA antibody (Ab7817, 1:80 dilution, Abcam; Cambridge, MA, USA). After repeated washing with phosphate-buffered saline, samples were incubated with a rabbit anti-mouse biotinylated secondary antibody. This was followed by avidin-biotin amplification with 3′-3′ dioaminobenzidine. Nuclear counterstain using Harris hematoxylin was followed by graded sequential dehydration in ethanol. Positive stain in bronchial and vessel walls was considered the internal control.

### Levels of TGF-β1 in bronchoalveolar lavage fluid (BALF)

BALF levels of transforming growth factor-β1 (TGF-β1) were measured in duplicate using available enzyme-linked immunosorbent assay kits (Quantikine; R&D Systems, Minneapolis, MN), according to manufacturer’s instructions, and microplates were read using a microplate reader (BIO-TEK Instruments, Winooski, VT).

### Western blot analysis of cleaved caspase-3 protein [19]

Equal amounts of proteins from lung homogenates were heat-denatured in Laemmli sample buffer with 2-mercaptoethanol (5%), resolved in 15% SDS-PAGE gel and transferred to nitrocellulose membranes (Thermoscientific, Rockford, IL, USA). Next, blots were blocked with 5% PBS-nonfat dry milk for 1 h at room temperature and then incubated with a rabbit monoclonal anti-active caspase-3 (cleaved) primary detection antibody (1:1000) (9661 Cell Signaling Technology, Danvers, MA, USA) overnight at 4 °C. After thoroughly washing with PBS 0.05% Tween-20, membranes were incubated for 2 h at room temperature with a goat anti-rabbit HRP-conjugated secondary antibody (1:5000) (Thermo Scientific, Rockford, IL). Cleaved caspase-3 immunoreactivity was visualized by enhanced chemiluminescence (SuperSignal™ Pico Chemiluminescent Substrate kit; Thermo Scientific, Rockford, IL, USA). C-DiGit Blot Scanner (Li-Cor, Lincoln, NE, USA) was used to image chemiluminescent signals by scanning. Densitometric analysis was performed using the ImageJ software version 1.46 m (NIH, Bethesda, MD). β-tubulin was used to control for equal loading.

### Co-immunofluorescence of cleaved caspase-3 and α-SMA proteins

Distribution of cleaved caspase-3 and α-SMA proteins was determined by co-immunofluorescence. Slides previously deparaffined and rehydrated were immersed in citrate buffer (0.02 M citric acid + 0.1 M sodium citrate) at 95 °C for 40 min. Non-specific reactivity was blocked with 10% fetal bovine serum for 10 min. Samples were then incubated with both a rabbit monoclonal anti-active caspase-3 (cleaved) primary detection antibody (9661, 1:300 dilution, Cell Signaling Technology, Danvers, MA, USA) and the mouse monoclonal anti-smooth muscle cell α-actin antibody (Ab7817, 1:300 dilution, Abcam; Cambridge, MA, USA) overnight at 4 °C. After repeated washing with PBS, samples were incubated with a goat anti-rabbit Alexa Fluor 488 conjugated antibody (DyLight™, Thermo Scientific; Rockford, IL, US) and with a goat anti-mouse Alexa Fluor 594 conjugated antibody (DyLight™, Thermo Scientific; Rockford, IL, US), for 2 h. DAPI (Thermo Scientific; Rockford, IL US) was used for nuclear staining. Images were obtained through the *Olympus FLUOVIEW* FV1000 confocal laser scanning microscope (Tokio, Japan) and processed using the ImageJ software, version 1.46 m (NIH, Bethesda, MD).

### TUNEL assay with myofibroblast co-localization

Lung tissue sections were treated by the fluorescein-in situ cell death detection kit (Roche, Manheim, Germany) for the detection of apoptotic cells, according to manufacturer’s instructions. Co-localization of TUNEL within myofibroblasts was performed using anti-α-SMA immunofluorescence. In brief, slides prepared for TUNEL were then incubated with the mouse monoclonal anti-smooth muscle cell α-actin antibody (Ab7817, Abcam; Cambridge, MA, USA) at a 1:300 dilution followed by incubation with a goat anti-mouse Alexa Fluor 594 conjugated secondary antibody (A-11032 DyLight™, Thermo Scientific; Rockford, IL, US), for 2 h. DAPI (Thermo Scientific; Rockford, IL US) was used for nuclear staining. Images were obtained through the *Olympus FLUOVIEW* FV1000 confocal laser scanning microscope (Tokio, Japan) and processed using the ImageJ software, version 1.46 m (NIH, Bethesda, MD).

### Lung tissue MMP-2 and MMP-9 activities

Gelatinolytic activity of lung tissue MMP-2 and MMP-9 was studied using zymography [20]. Thirty μg of total protein in lung tissue homogenate were loaded into a gelatin-containing electrophoresis gel (10% polyacrylamide and 1% gelatin under non-reducing conditions). After electrophoresis, gels were washed in 2.5% TritonX-100 (Sigma-Aldrich, St. Louis, MO) to remove SDS, incubated overnight at 37 °C in a calcium containing developing buffer, stained with 0.1% Coomassie Brilliant Blue and destained until areas of gelatinolytic activity became evident. Densitometric analysis was performed using ImageJ software version 1.46 m (NIH, Bethesda, MD).

### Western blot analysis of TIMP-2

15% SDS-PAGE was utilized and membranes were probed with an anti TIMP-2 antibody (AB770, 1:1000 dilution, Merck KGaA, Darmstadt Germany) and then, with a goat anti-rabbit HRP-conjugated secondary antibody (Thermo Scientific, Rockford, IL). TIMP-2 immunoreactivity was visualized by enhanced chemiluminescence (SuperSignal™ Pico Chemiluminescent Substrate kit; Thermo Scientific, Rockford, IL, USA). C-DiGit Blot Scanner (Li-Cor, Lincoln, NE, USA) was used to image chemiluminescent signals by scanning. Densitometric analysis was performed using the ImageJ software version 1.46 m (NIH, Bethesda, MD). β-tubulin was used to control for equal loading.

### Statistical analysis

Non-parametric analysis of variance (Kruskall-Wallis) and Spearman’s rank correlation were used [21]. A *P* value < 0.05 was considered statistically significant. Analyses were done using GraphPad Prism 5.0 software.

## Results

### Resolution of lung injury after a single instillation of gastric fluid

**Fig. 2:**
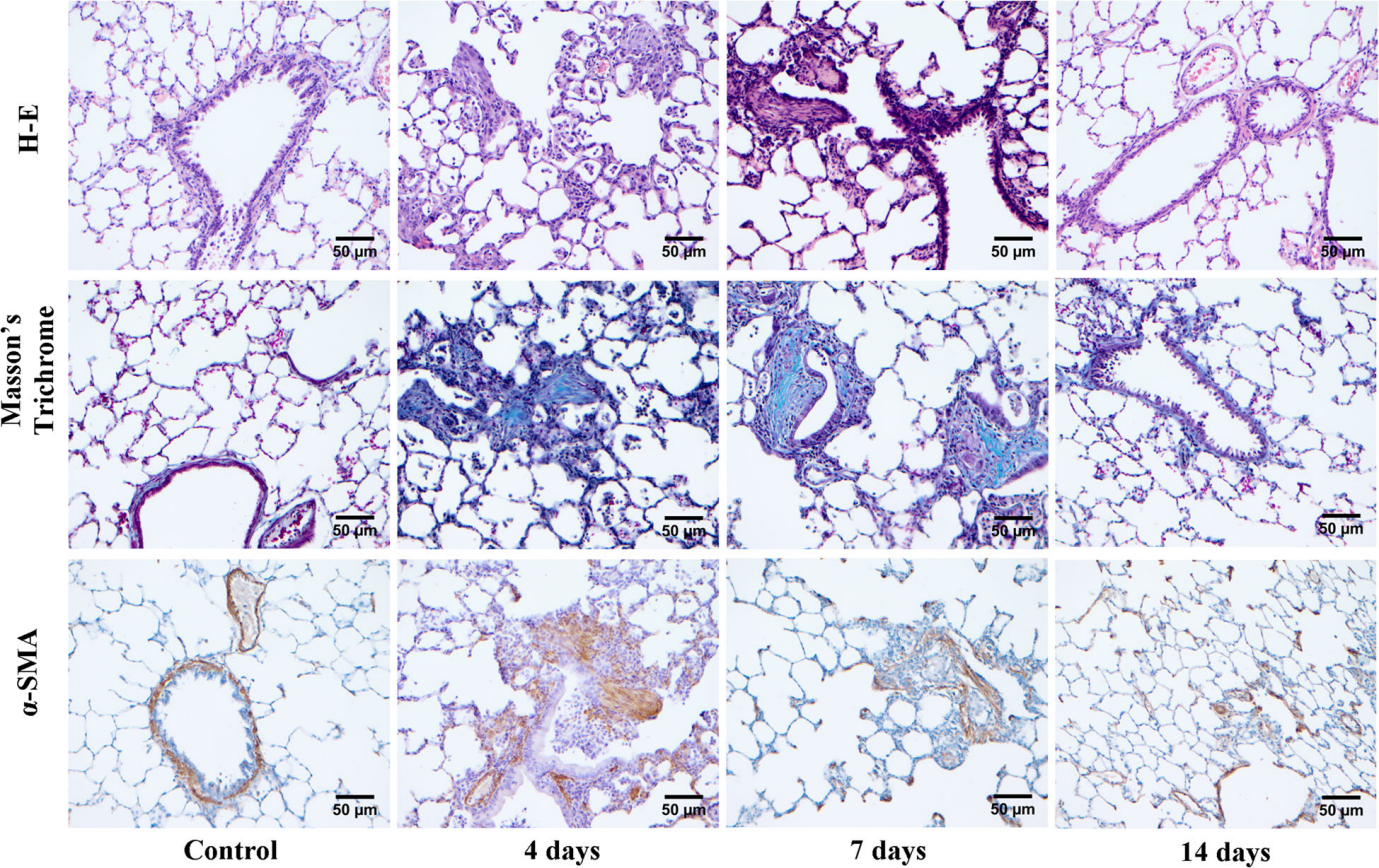
Resolution of lung injury after a single instillation of gastric fluid. Light microscopy (hematoxylin-eosin, Masson’s trichrome and α-SMA immunohistochemistry) of rat lung at days 4, 7 and 14 after a single orotracheal instillation of gastric fluid**.** With H-E (upper panel), intra-alveolar buds of granulation tissue (Masson bodies), characteristic of OP at days 4 and 7 are observed. Foreign body giant cells, either isolated or forming granulomas are also seen. This pattern is no longer present at day 14. With trichrome stain (middle panel) and α-SMA immunostaining (lower panel) abundant collagen fibers are seen intermixed with myofibroblasts in Masson bodies at days 4 and 7. These findings are no longer present at day 14. Original magnification: 200X

Figure 2 illustrates histological changes at days 4, 7 and 14 after instillation of gastric fluid using H-E and trichrome staining as well as α-SMA immunohistochemistry. H-E stain shows abundant intra-alveolar buds of granulation tissue, characteristic of organizing pneumonia at days 4 and 7 that are no longer present at day 14. Foreign body giant cells, either isolated or forming granulomas are frequently observed at days 4 and 7 after instillation, but not at day 14. Markers of ALI are not observed.

Trichrome staining and α-SMA immunostaining show intra-alveolar buds of collagen surrounded by myofibroblasts at days 4 and 7 that disappear at day 14. The mean area of α-SMA -positive stain in Masson bodies showed a progressive reduction between days 4 and 14 after instillations (r_(Spearman)_: − 0.8041; *p* < 0.05).

### Evidence of apoptosis during injury resolution after a single instillation of gastric fluid

#### Caspase-3 activation

**Fig. 3:**
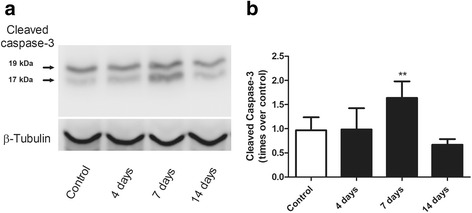
Caspase-3 activation during injury resolution after a single instillation of gastric fluid. **a** Representative Western blot of lung tissue homogenate illustrating the time course of changes in cleaved caspase-3 expression. This immunoblot is from a single set of animals (one animal sample per time point) and is representative of 6 separate sets. The immunoblot shows that the 19/17 kDa bands seen in the control sample show no change at day 4, a significant enlargement at day 7 and are not different from the control bands at day 14. β-tubulin immunoblot shows equal protein loading. **b** Densitometric analysis of six independent Western blots expressed as times over control, showing a significant 60% increase in cleaved caspase-3 at day 7 only. Western blot bands were normalized to beta-tubulin. Data are means ± SD. **: *p* < 0.01 with respect to controls

Figure 3 illustrates changes in lung tissue homogenate caspase-3 activation using Western blot analysis of the 19/17 kDa active caspase-3 bands after a single instillation of gastric fluid. In Fig. 3a, the 19/17 kDa immunoreactive bands seen in the control sample show no change at day 4, a significant enlargement at day 7 and, are similar to the control bands at day 14. This immunoblot is from a single set of animals (one animal sample per time point) and is representative of 6 separate sets. Figure 3b illustrates the densitometric analysis of six Western blots for cleaved caspase-3 showing no change at day 4 and a mean 60% increase in immunoreactivity at day 7 (*p* < 0.01).

#### Cleaved caspase-3 location

**Fig. 4:**
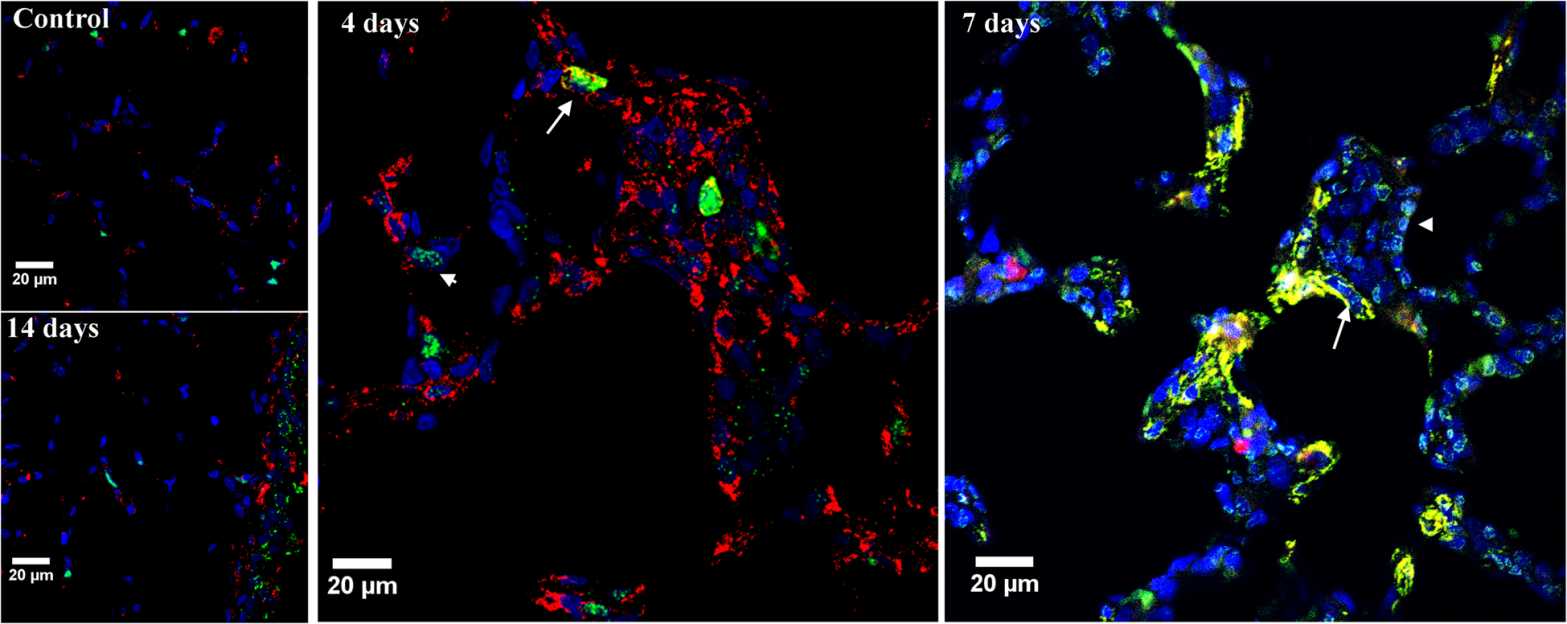
Cleaved caspase-3 location in intra-alveolar buds of granulation tissue after a single gastric fluid instillation. Representative images of indirect immunofluorescence of normal lung and of lung studied at days 4, 7 and 14 after a single orotracheal instillation of gastric fluid using antibodies to cleaved caspase-3 (green) and α-SMA (red) as primary antibodies. Cell nuclei are co-stained (blue) with DAPI (4′,6-diamidino-2-phenylindole). Evidence of caspase-3 activation is present in intra-alveolar bud-α-SMA –positive and –negative cells at days 4 and 7 only. Arrows indicate α-SMA-positive cells that are also positive for cleaved caspase-3. Arrowheads indicate α-SMA-negative cells that are positive for cleaved caspase-3. Original magnification 630X

Figure 4 illustrates the results of the indirect immunofluorescence study of lung tissue at days 4, 7 and 14 after a single orotracheal instillation of gastric fluid using antibodies to cleaved caspase-3 and α-SMA as primary antibodies, in order to elucidate which of the many cells present in intra-alveolar fibrosis undergo apoptosis. This figure is representative of three sets of animals. Results show that caspase-3 activation is present in many cells that are part of the intra-alveolar buds at days 4 and 7 only. Some of these cells are myofibroblasts since they are α-SMA –positive. However, Fig. 4 also shows α-SMA –negative cells exhibiting caspase-3 activation. Giant cells as well as histiocytes, lymphocytes and epithelial cells, that are part of this injury stage [7], could be involved.

#### Detection of apoptosis by TUNEL assay

**Fig. 5:**
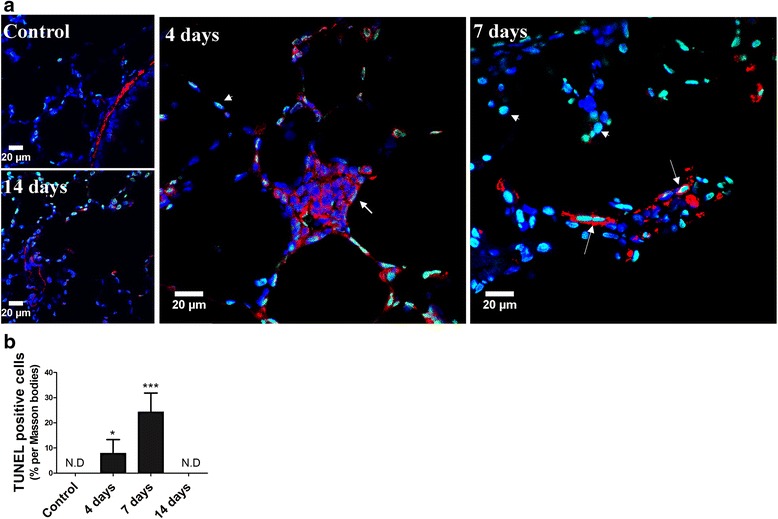
Detection of apoptosis by TUNEL assay in intra-alveolar buds of granulation tissue. **a** Representative images of double immunofluorescence of TUNEL (green) and α-SMA (red) in normal lung and in lung studied at days 4, 7 and 14 after a single orotracheal instillation of gastric fluid. Cell nuclei are co-stained (blue) with DAPI (4′,6-diamidino-2-phenylindole). Apoptosis is executed in α-SMA –positive and –negative cells inside intra-alveolar buds at day 4 and more intensely at day 7 after gastric fluid instillation. Arrows indicate α-SMA-positive cells that are also positive for TUNEL. Arrowheads indicate α-SMA-negative cells that are TUNEL positive. Original magnification: 630×. **b** Quantitation of TUNEL positive cells in percentage of total cells in Masson bodies. *: *p* < 0.05; ***:*p* < 0.001 in comparison to controls. ND: non detectable

Figure 5 shows the results of the double immunofluorescence studies for TUNEL and α-SMA. Figure 5a shows that apoptosis is executed in α-SMA –positive and –negative cells inside intra-alveolar buds at day 4 and more intensely, at day 7 after instillation. There is no evidence of apoptosis at day 14. Figure 5b shows quantitation of TUNEL positive cells in percentage of total cells. TUNEL positive cells in Masoon bodies (6 to 7 Masson bodies/slide) are: 7.8% at day 4 (*p* < 0.05), 24.2% at day 7 (*p* < 0.001) and are not detected at day 14.

#### Lack of elevated levels of TGF-β1 in the lung during myofibroblast apoptosis execution

**Fig. 6:**
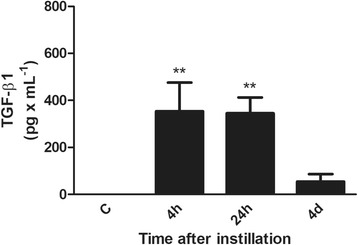
Changes in TGF-β1 levels in BALF. TGF-β1 levels show a significant increase very early during acute lung injury, starting at 4 h, remaining elevated for 24 h and reaching control (C) values at day 4. Data are means ± SEM. **p < 0.01

Figure 6 shows elevated levels of TGF-β1 in BALF during the initial acute lung injury phase. They increase significantly in coincidence with elevation in the levels of several pro-inflammatory cytokines (data not shown) during the first 24 h after instillation but are back to control values at day 4.

### Activity of MMP-2 and expression of TIMP-2 after a single instillation of gastric fluid

**Fig. 7:**
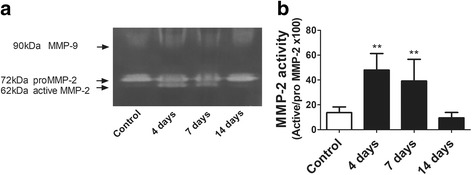
Lung tissue MMP-2 activity after a single instillation of gastric fluid. **a** Representative zymogram of lung tissue homogenate showing the time course of changes in MMP-2 activity. No signal is seen for MMP-9. This zymogram is from a single set of animals (one animal sample per time point) and is representative of 4 separate sets. It shows a 62-kDa gelatinolytic band density (corresponding to the full-active MMP-2) at day 4 and 7 after instillation that is not present in the control sample or in the day 14 sample. The 72 kDa gelatinolytic band density corresponding to the pro-MMP2 remains stable in size and not different from the control band at the time points studied. **b** Densitometric analysis of four independent zymograms for MMP-2 activity. The ratio of full active MMP-2 (62-kDa) to inactive pro-MMP-2 (72 kDa) in percentage is shown. This ratio is significantly increased at days 4 and 7 and is similar to controls at day 14. Data represent the means ± SD. **: *p* < 0.01 with respect to controls

Figure 7 shows changes in lung tissue MMP-2 activity after a single instillation of gastric fluid. In Fig. 7a, a representative zymogram is shown, with no MMP-9 signal, but with significant changes in band densities corresponding to MMP-2. This zymogram is from a single set of animals (one animal sample per time point) and is representative of 4 separate sets. It shows a 62-kDa gelatinolytic band density (corresponding to the full-active MMP-2) at day 4 and 7 after instillation that is not present in the control sample or in the day 14 sample On the other hand, the 72 kDa gelatinolytic band density corresponding to the pro-MMP2 remains stable in size and not different from the control band during the time of the study. The densitometric analysis in Fig. 7b shows the ratio of full active MMP-2 (62-kDa) to inactive pro-MMP-2 (72 kDa) as a percentage, that increases 3.5 times at days 4 and 7 with respect to controls (*p* < 0.01) and is not significantly different from controls at day 14.

**Fig. 8:**
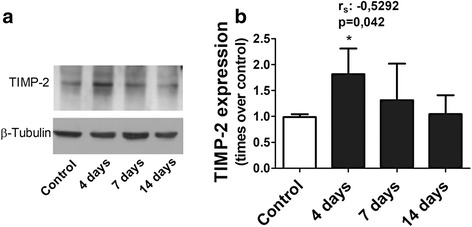
Lung tissue TIMP-2 expression by Western blot after a single instillation of gastric fluid. **a** Representative Western blot of lung tissue homogenate illustrating the time course of changes in TIMP-2 expression. This immunoblot is from a single set of animals (one animal sample per time point) and is representative of 5 separate sets. The approximately 30 kDa band density corresponding to TIMP-2 seen in the control sample increases significantly at day 4 and is similar to the control band at days 7 and 14. β-tubulin immunoblot shows equal protein loading. **b** Densitometric analysis of five independent Western blots showing a significant progressive reduction in TIMP-2 after a significant increment at day 4. Western blot bands were normalized to beta-tubulin. Data are means ± SD. *: *p* < 0.05 with respect to controls. r_S=_ Spearman rank correlation coefficient for the reduction in band size from days 4 to 14

Figure 8 shows changes in TIMP-2 expression in lung tissue homogenate after a single instillation of gastric fluid. The representative Western blot shown in Fig. 8a illustrates that the approximately 30 kDa band density corresponding to TIMP-2 seen in the control sample is increased at day 4 and similar to the control band at days 7 and 14. This immunoblot is from a single set of animals (one animal sample per time point) and is representative of 5 separate sets. Figure 8b illustrates the densitometric analysis of five Western blots showing a significant 80% increment in TIMP-2 expression at day 4 (*p* < 0.05) that is followed by a significant progressive reduction in TIMP-2 expression (r_(Spearman):_ − 0.5292; *p* = 0.042).

## Discussion

In the model of aspiration-induced lung injury the repair process involves an intra-alveolar fibroproliferative response with the characteristics of organizing pneumonia that requires elimination for normal lung function restoration. The possibility that cell death accounts for the regulation of myofibroblast number in the OP developing after lung injury by gastric fluid has never been demonstrated. In this study we tested the hypothesis that apoptosis participates in this process. Our results clearly show that activation of apoptosis during the repair phase of aspiration-induced ALI contributes to the disappearance of cells that are characteristics of this fibroproliferative response. Both myofibroblasts and other cells that are part of this response exhibit evidence of apoptosis. At the peak of apoptosis, an imbalance between MMP-2 activity and TIMP-2 expression is also established by reduction in TIMP-2 expression, contributing to the removal of non-cellular components of the fibroproliferative response.

We have previously shown [7] that the consequences of a single event of aspiration in the rat are manifested by a continuum of alterations starting with severe ALI and evolving toward a fibroproliferative response that exhibits most of the constitutive elements of OP such as intraluminal plugs of myofibroblasts and collagen fibers affecting small bronchioles, alveolar ducts, and peribronchiolar alveolar spaces [22]. We have also shown that this pattern is reversible, leaving the lung with normal architecture [7]. Mechanisms involved in cellular and extracellular matrix components disappearance during repair have not been studied in this model.

The continuum of alterations described in the rat model shares significant similarities with the phases described in normal cutaneous wound healing [8, 9]. Evidence suggests that during the various stages of wound healing, apoptosis is the main cause of decreasing cellularity [23]. Whereas in the early phases apoptosis affects inflammatory cells as early as 12 h after wounding [24], myofibroblast apoptosis begins several days later, after the acute inflammation has resolved and at about the same time as wound closure [11]. Thus, apoptosis is considered to be the primary mechanism of myofibroblast elimination in granulation tissue during the resolution phase of normal wound repair.

With regard to ALI, apoptosis has been studied mainly in the early stages, due to the fact that most models of ALI are not long enough to study the repair phase [3, 5, 6]. Thus, several investigators have shown apoptosis of epithelial [12, 17] and inflammatory cells [13, 17] in the early stages. Epithelial injury in ALI has been shown to be associated with apoptotic death of alveolar epithelial cells triggered by soluble mediators [12, 13, 17]. Other possible pathways have also been explored [4, 13].

Compared to the role of apoptosis in the early phase of ALI, its role in the remodeling/repair phase is less well understood. There is very limited data that apoptosis of lung and inflammatory cells participate in the resolution of ALI and none regarding aspiration-induced ALI. Studies have mainly focused on apoptosis of type II alveolar epithelial cells [14] showing that this process occurs as a normal response in the organizing phase of ALI. With regard to studies focusing on fibroblasts apoptosis during injury repair, Polunovsky et al. [25] showed that BALF from patients in the repair phase of ALI induces death of fibroblasts in vitro by a mechanism similar to apoptosis; in addition they found evidence of apoptosis in intra-alveolar granulation tissue in biopsies from patients recovering from ALI, but did not provide information about the cell types involved. Apoptosis by dysruption of adhesion of myofibroblasts triggered by soluble synthetic fibronectin peptides was shown in vitro by Hadden et al. [26].

In our model, resolution of the fibroproliferative response starts early, with histological evidence of reduction in cellularity and in ECM components between days 4 and 7 to significantly resolve at day 14. Evidence of apoptosis was found in myofibroblasts as well as in other cell types that in this model involve foreign body giant cells, histiocytes and lymphocytes, preceding the reduction in cell number and ECM components. Thus, apoptosis of myofibroblasts and other cell types in the Masson body is probably a normal physiological mechanism involved in the resolution of intra-alveolar fibrosis in our model.

Lappi-Blanco et al. [27] in a comparative study of lung biopsies of patients with bronchiolitis obliterans organizing pneumonia (BOOP) and usual interstitial pneumonia (UIP) showed differences in the number of apoptotic events, with BOOP exhibiting significantly more events affecting several cell types, among them myofibroblasts. They postulated that apoptosis is involved in BOOP because of the more reversible character of this condition compared with UIP. Our results in the animal model help in understanding the results of Lappi-Blanco et al. that are limited by the snap-shot character of human biopsies, by contextualizing the evidence of apoptosis as part of the resolution and repair process in OP in humans.

It has been widely described both *in vivo* and *in vitro* that the myofibroblast is an apoptosis resistant-cell type in many pathological conditions that are characterized by persistent fibrosis [27–29]. However, there is limited data on apoptosis sensitivity of myofibroblasts under normal repair conditions of the lung. Mechanical resistance of the ECM and the presence of TGF-β1, an important pro-fibrotic cytokine, are known to be minimal conditions for myofibroblast differentiation [29]. In this sense, our results showed that in the early phase of acute lung injury (24 h) there is an increase in the levels of TGF-β1 in BALF that return to control values at day 4, time in which we observed the first evidence of apoptosis of myofibroblasts in our model. Taking these results together, our data suggests that the lack of persistence of TGF-β1 in lung tissue during the repair phase of ALI could be one of the key conditions to initiate myofibroblast apoptosis and thus, resolution of the fibroproliferative response in our model. Supporting this interpretation, *in vitro* studies have shown that the persistence of TGF-β1 expression, promotes a pro-survival/anti-apoptotic phenotype in myofibroblasts when exposed to typical pro-apoptotic stimuli [30, 31].

### ECM synthesis/degradation imbalance associated to apoptosis during repair of aspiration-induced ALI

**Fig. 9:**
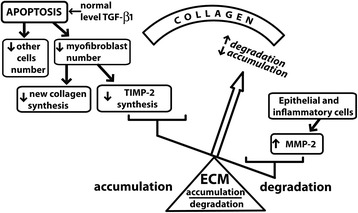
Apoptosis-related ECM synthesis/degradation imbalance. Apoptosis is involved in the repair phase of gastric-fluid induced ALI, affecting myofibroblasts among other mesenchymal cells that are part of intra-alveolar buds of granulation tissue. Reduction in myofibroblast number results in reduction of new collagen synthesis. Since myofibroblasts are also an important source of TIMP-2, synthesis of this inhibitor is also reduced, leaving increased MMP-2 activity unbalanced and shifting the ECM synthesis/degradation balance toward degradation of pre-formed ECM

Loss of collagen-producing myofibroblasts is not sufficient for adequate intra-alveolar fibrosis resolution, since degradation of the ECM is also required. Whether resolution of already deposited non-cellular components of intra-alveolar fibrosis can be related to apoptosis of cellular components is not known*.* In vitro studies have shown that apoptosis signals could be involved in the regulation of collagen degradation by inducing MMP-2 expression. In this regard, Bian and Sun described a p53–dependent transcriptional activation of the promoter of the human MMP-2 gene [32], suggesting that apoptosis signals may participate in down-regulation of collagen deposition by both decreasing myofibroblast number and, by activating MMP-2. To gain insight into the possibility that apoptotic changes could be associated with ECM remodeling in the repair phase of our model, we studied changes in MMP-2 activity and TIMP-2 expression. Our results show that at day 4, prior to the peak in myofibroblast apoptosis, there is increased MMP-2 activity that is likely balanced by increased TIMP-2 expression. However, at day 7, at the peak of apoptosis, MMP-2 activity remains elevated whereas TIMP-2 expression is reduced, likely contributing to a shift in the balance between MMPs and TIMPs toward an increase in ECM degrading activity. This result provides evidence for ECM remodeling in relation to myofibroblast apoptosis, since the myofibroblast is one of the sources of TIMP-2 [33]. Similar results in experimental liver fibrosis have been shown by Iredale et al. [34] who demonstrated that during fibrosis resolution, there is a rapid decline in TIMP levels, shifting the overall MMPs–TIMPs balance, thus resulting in increased ECM degrading activity. In addition, in vitro studies by Hartland et al. [35] have shown that MMP-2 can induce apoptosis in hepatic stellate cells concomitant with degradation of N-cadherin, suggesting that MMP-2 may enhance the resolution of liver fibrosis by triggering hepatic stellate cell apoptosis. In this context, several mechanisms are likely to contribute to the shift in the ECM synthesis/degradation balance toward degradation in our model, among them the increment in MMP-2 activity that favors collagen degradation and triggers myofibroblast apoptosis and, the reduction in TIMP-2 production by myofibroblat apoptosis, that leaves MMP-2 activity unbalanced. Figure 9 summarizes the mechanisms that we propose play a role in the resolution of intra-alveolar fibrosis in this model. It involves apoptosis of myofibroblasts that results in ECM synthesis/degradation imbalance.

We believe that our results showing how resolution mechanisms work during normal lung repair after a single and limited injury by gastric fluid, shed light to better understand the resolution phase of ALI induced by other non-persistent injuries. It remains to be studied if the resolution pathway that we are describing with a single and limited injury participates in a similar way or instead, it becomes dysregulated favoring non-reversible fibrosis when there is persistence of the injurious agent.

Factors that regulate myofibroblat apoptosis remain to be elucidated in this model. Our experimental design was not intended to study regulation of myofibroblast apoptosis. Nevertheless we identified some possible conditions that could be related to apoptosis initiation, such as normal levels of TGF-β1 (after initial elevation) and activation of MMP-2 preceding apoptosis. Future studies into the physiological triggers for myofibroblast apoptosis are warranted. Without inhibitors of either apoptosis or MMPs it is difficult to know whether activation of these mechanisms could be considered the cause of lung injury resolution in our model**.** Future studies could explore whether cell apoptosis will be inhibited in genetically modified mice with constitutively activated TGF-β1 signaling pathway. On the other hand, abrogation of apoptosis and/or MMP-2 activity during the subacute phase of gastric fluid-induced ALI could be useful to determine whether injury resolution will not occur.

## Conclusions

The model of severe ALI by a single instillation of gastric fluid that ends in restitution of normal lung architecture provides a great tool to identify pathways involved in lung injury resolution, reducing the existing gaps in understanding lung fibrosis resolution. We have clearly shown that cell death accounts for the regulation of myofibroblast number in the OP developing after lung injury by gastric fluid. Apoptosis is activated in alpha-SMA-positive and –negative cells forming part of the intra-alveolar buds of fibrosis during the repair phase of ALI in this model. Apoptosis of myofibroblasts, by reducing new collagen synthesis and TIMP-2 production, plays a significant role in the resolution of OP induced by gastric fluid aspiration.

## Abbreviations

ALI: Acute lung injury
BALF: Bronchoalveolar lavage fluid
BOOP: Bronchiolitis obliterans organizing pneumonia
ECM: Extracellular matrix
MMP-2: Matrix metalloproteinase-2
OP: Organizing pneumonia
TGF-B1: Transforming growth factor beta1
TIMP-2: Tissue inhibitor of metalloproteinase-2
TUNEL: Terminal deoxynucleotidyl transferase (TdT)-mediated deoxyuridine triphosphate nick-end labeling
UIP: Usual interstitial pneumonia
α-SMA: Alpha-smooth muscle actin
